# Food-borne disease and climate change in the United Kingdom

**DOI:** 10.1186/s12940-017-0327-0

**Published:** 2017-12-05

**Authors:** Iain R. Lake

**Affiliations:** School of Environmental Sciences, University of East Anglia, Norwich, NR4 7TJ UK

**Keywords:** Climate change, Food-borne disease, Adaptation, Salmonella, Campylobacter, Gastrointestinal infections, Global warming

## Abstract

This review examined the likely impact of climate change upon food-borne disease in the UK using *Campylobacter* and *Salmonella* as example organisms. *Campylobacter* is an important food-borne disease and an increasing public health threat. There is a reasonable evidence base that the environment and weather play a role in its transmission to humans. However, uncertainty as to the precise mechanisms through which weather affects disease, make it difficult to assess the likely impact of climate change. There are strong positive associations between *Salmonella* cases and ambient temperature, and a clear understanding of the mechanisms behind this. However, because the incidence of *Salmonella* disease is declining in the UK, any climate change increases are likely to be small. For both *Salmonella* and *Campylobacter* the disease incidence is greatest in older adults and young children. There are many pathways through which climate change may affect food but only a few of these have been rigorously examined. This provides a high degree of uncertainty as to what the impacts of climate change will be. Food is highly controlled at the National and EU level. This provides the UK with resilience to climate change as well as potential to adapt to its consequences but it is unknown whether these are sufficient in the context of a changing climate.

**Electronic supplementary material:**

The online version of this article (10.1186/s12940-017-0327-0) contains supplementary material, which is available to authorized users.

## Background

Climate change may have many impacts upon the weather and climate of the United Kingdom [1], and in this paper we focus upon its potential effects on food. We focus initially upon two food-borne diseases, *Campylobacter* and *Salmonella*. These are chosen because, in addition to their public heath importance, there is much evidence that they are influenced by existing climate variability especially temperature [2]. Therefore, under a warmer climate, incidence of these infections may change. An understanding of how these two food-borne diseases may be affected by climate change provides important insight into what the impacts may be on other food-borne diseases. The purpose of this review is to consider what the likely impacts of climate change will be, as well as to consider the distributional impacts of any changes. In addition, the review will also consider in less detail other potential impacts which are less well documented in the literature. Due to the broad nature of this topic, this review was not systematic review but built upon 2 previous reviews [1, 3].

Although the geographical focus of this review is the UK, international borders can be crossed by food-borne disease implying that changes in foodborne disease in one country may have consequences in others. For example, of the infectious intestinal disease recorded in the UK (of which food-borne disease is a subset) 8–12% are estimated to have been caught overseas [4]. Furthermore the food supply chain is global and so any impacts of the food supply chain in one country can have impacts elsewhere. Only 53% of the total food consumed in Britain is home grown [5]. Food and drink are also important export markets for the UK and so climate change induced food safety changes in the UK could have global consequences.

### The evidence; *Campylobacter*

In developed countries, including the UK, *Campylobacter* is the most common bacterial cause of diarrhoeal disease. It can cause abdominal pain and severe diarrhoea. Clinical complications include Guillain-Barre syndrome which requires intensive care in some 20% of cases, and can be fatal [6]. Although poultry consumption is widely implicated as a source of *Campylobacter* many other factors are thought to play a role and many features of the disease are difficult to explain (e.g. spring peak). Consequently the epidemiology of *Campylobacter* is complicated [7] and the transmission pathways for a large proportion of cases are unknown [8]. In terms of UK health outcomes following *Campylobacter* infection a recent study estimates that *Campylobacter* is the major bacterial Infectious Intestinal Disease agent in the UK, leading to over 500,000 cases and 80,000 consultations to general practice annually [9]. In 2008 the annual cost of acute *Campylobacter* infection was estimated to be £600 million for England and Wales.Reported *Campylobacter* disease also appears to be increasing [10–12]. These increases have been occurring in spite of biosecurity initiatives to exclude *Campylobacter* from poultry flocks.

*Campylobacter* shows a strong seasonal variability leading researchers to believe that it may be affected by climate change. This is coupled with numerous studies indicating that *Campylobacter* infections are associated with climate variability. The most commonly reported factor is a positive association with temperature [2, 13, 14]. However, our understanding of the reasons behind this are limited because unlike other bacteria *Campylobacter* does not multiple outside the gut. For example the response of *Campylobacter* cases to season and weather patterns has been attributed in the literature to several factors such as the cycling of the organisms in natural reservoirs and the seasonality of countryside visits exposing the public to *Campylobacter* in the environment [15]. Other authors have suggested the importance of seasonal and weather associated changes to food consumption (e.g. barbecuing [7], elevated consumption of fruit and salad increasing risk of cross-contamination [2]). *Campylobacter* transmission to humans is complex ecologically with multiple hosts and transmission pathways, and currently is poorly understood.

In terms of where disease levels are highest, elevated incidence in rural areas is a common finding in many [16, 17] but not all studies [18]. In England and Wales the highest incidence is found in rural areas [7]. In Scotland this rural excess was only observed in the under 5 s [19]. Strachan et al. [19] were able to attribute *Campylobacter* infections to different sources using Multilocus Sequence Typing. They argue that the major source of infection for young children in urban areas is chicken, whereas for rural children ruminant and other avian sources are of elevated importance.

Studies across the UK indicate that disease incidence is higher in less deprived areas [7, 20], although because these studies are based upon reported cases of *Campylobacte*r, some differences may be due to differential reporting [21]. Gillespie et al. [22] found in England and Wales that *Campylobacte*r disease incidence was slightly higher in individual’s whose work was often done in an office or other professional environment in comparison to those whose jobs were more manual. However, disease incidence was highest in people working in semi-routine occupations [23]. This same study found that the incidence of *Campylobacter* disease was greatest in the Pakistani population in comparison to the white population. Levels in other ethnic groups such as Indian, Black and Chinese were lower. Turning to gender, this study found that the incidence of disease was slightly higher in males than in females, a result confirmed in Scotland [24]. In terms of the age distribution of reported cases the greatest incidence appears to fall on infants. Incidence then decreases for the ages 2–13 years but rises again until age 22. Incidence then remains relatively constant between ages 22 and 69 before falling from age 70 onwards [22]. Similar distributions are reported in Scotland and Northern Ireland [12]. In terms of trends over time, as the UK population ages the number of reported *Campylobacter* cases has increased in older individuals. However, as well as the absolute number of reports increasing it has also been observed that *Campylobacter* incidence is increasing in older people [7].

### The evidence; *Salmonella*

Infection with *Salmonella* leads to diarrhoea, fever and abdominal cramps, usually 1–3 days after the initial infection. Symptoms generally last for 4–6 days but in some individuals the patient may need to be hospitalised. Although there are a number of potential pathways of transmission for *Salmonella*, the consumption of raw or undercooked eggs or poultry are recognised to be of major importance. Several thousand *Salmonella* species (serotypes) have been identified and these have differing routes of transmission. For example *Salmonella* Enteritidis is commonly associated with eggs whereas *Salmonella* Typhimurium is associated with a wider variety of foods [2]. A recent study in England estimates that there are just under 39,000 cases of *Salmonella* a year leading to just over 11,000 GP consultations [9]. This is a large reduction in cases in comparison to the early 1990’s. In contrast to *Campylobacter*, *Salmonella* outbreaks are common and so as a disease it is likely to be prominent in public consciousness. Nonetheless *Salmonella* is not a priority pathogen identified by the Food Standards Agency for specific action [25]. Older research focusing upon England and Wales at a time when levels of *Salmonella* disease was higher, estimated that in 2000 it led to over 8500 hospital admissions and 119 deaths NB more estimated deaths than Campylobacter: [26]. It has been estimated that the average cost of a *Salmonella* case is around £1000 [27]. Multiplying this by the estimated community cases produces a total UK cost of £39million p/a (This assumes that the costs of reported and non-reported cases are similar and so is probably an overestimate).

Salmonella is climate sensitive and infections are more frequent in summer. Stronger evidence emerges from studies indicating that in warm weather Salmonella infections are elevated [14, 28]. Furthermore, there is a clear biological understanding of the mechanisms involved as Salmonella can grow in food kept at ambient temperature [29]. Therefore in a warmer world, Salmonella infections could increase. Across Europe the numbers of cases are currently declining because intervention has proved effective through the vaccination of animals, increased biosecurity and slaughtering out.

In terms of highlighting whether levels of illness with *Salmonella* is higher in rural or urban areas no UK studies have been conducted. No difference has been found in studies in the USA, Germany and France [30–32]. A New Zealand found higher disease incidence in rural areas [33]. This lack of association is backed up by recent microbiological work suggesting that local domestic animals (e.g. cows and sheep) are not a major source of *Salmonella* in humans [34]. There are also no UK studies examining which socioeconomic groups are most affected. US studies have found lower disease incidence in areas with poorer educational attainment [35, 36]. However, this contradicts a Canadian study [37]. There are no UK studies examining differentiation between ethnic groups, but in the US minority populations tend to suffer disproportionally [36, 38]. In terms of the age distribution of cases in England the reported highest incidence of disease was is in the under 4 s reducing until age 14. From this point incidence is fairly constant.Similar age distributions are reported in Scotland [39] and Northern Ireland [12]. The increasing use of proton pump inhibitors may increase susceptibility to *Salmonella* [40] and the use of these in older populations is increasing.

### Climate change impacts; *Campylobacter*

This review has presented evidence that illness with *Campylobacter* is associated with weather; disease incidence is greater in the summer and during periods of warmer weather incidence is also elevated. Therefore, it would seem logical to assume that climate change would have an impact upon this disease. Although European Infectious Disease experts share a broad agreement that climate change will impact upon *Campylobacter*, this is not the case in the UK [41]. However, this is at odds with other UK sources which do suggest a moderate impact. This ambiguity may be due to uncertainty over the exact pathways through which weather affects disease incidence. Weather may be associated with *Campylobacter* but we are unsure as to why. Outside the UK there are projections of changes in *Campylobacter* as a result of climate change. Cullen [42] projects increases in *Campylobacter* in Ireland of between 2 and 3%. A study in Montreal forecasts that by 2055, *Campylobacter* could increase 23% [43]. However, given that such studies effectively treat the mechanisms involved as a “black box” it could be argued that these projections are highly uncertain.

Schijven et al., examines the use of a decision support tool for determining the links between *Campylobacter* and climate change. Instead of examining associations between weather and *Campylobacter* they use a Quantitative Microbial Risk Assessment approach and split their analysis into a number of pathogen pathways (drinking water, bathing water, oysters and chicken fillet). Within each pathway a number of models are used to estimate climate change impacts. The results indicate that *Campylobacter* cases associated with poultry consumption are likely to increase under climate change whereas risks associated with the drinking water pathway are likely to decrease due to increased inactivation in higher warmer temperatures.

### Climate change impacts; *Salmonella*

There are strong links between *Salmonella* and the environment especially ambient temperature. However, in contrast to *Campylobacter* there is a much clearer biological mechanism explaining why higher temperature leads to an elevated incidence of illness with *Salmonella*. At elevated ambient temperatures *Salmonella* reproduction is enhanced. However, in spite of this biological mechanism, UK Infectious Disease experts still do not consider *Salmonella* to be one of the diseases most likely to be affected by climate change [41]. This may be because control measures appear to have substantially reduced levels of the disease since the early 1990’s to the point where it is not considered a priority pathogen. There is further evidence that over time the UK is becoming increasingly tolerant to the effects of temperature upon Salmonella infections.However, it is worth considering that around a quarter of Salmonella cases are associated with foreign travel [44], of which the nation has little influence.

Globally there have been some attempts to model future *Salmonella* changes. A recent Australian study estimated by 2050 an extra 4000–7000 *Salmonella* cases annually [45]. A second Australian study found that, assuming that all other factors remain constant, salmonellosis might increase 56% by 2050 in South Australia [46]. A recent European study indicated that under the climate change A1B scenario, the number of *Salmonella* cases could increase 9.3–16.9% by the 2080’s depending upon the level of mitigation. No specific details are provided for the UK although the study highlights the UK as a country where the largest increase in cases occurs.

### Climate change; other potential impacts

Other intestinal infectious diseases vary seasonally or are sensitive to weather. Consequently climate change could affect such diseases. However, currently there is a lack of evidence on which organisms are likely to be affected and what the public health importance of these are. There are also many different mechanisms through which pathogen prevalence changes could occur, such as changing animal husbandry affecting animal to animal transmission, or new weather patterns altering the survival of pathogens in the environment [47]. Therefore, identifying systems and pathogens most likely to be affected is nearly impossible [47]. It is suggested that pathogens with low infective doses are most likely to be affected by climate change (e.g. enteric viruses, *Shigella* spp., enterohemorrhagic *E. coli* strains and parasitic protozoa). Those with significant environmental persistence (e.g. enteric viruses and parasitic protozoa) are also likely to be most affected alongside pathogens with recognised stress tolerance responses to pH and temperature (e.g. enterohemorrhagic *E. coli* and *Salmonella*).

In addition to infectious intestinal disease climate change may have other impacts on food. For example within agriculture one impact may be changes to the seasonal patterns and abundances of pest species and plant diseases both in the UK and globally. Boxall et al., [48] highlight that these changes will lead farmers to alter their use of herbicides, pesticides [49] and fungicides in response. This may alter the levels of these residues in food. In addition to changing farming practices, climate change may also affect the transport of food contaminants. Changing soil properties may affect the bioavailability of heavy metals [48], while more extreme weather could increase the transport of contaminants by flooding [50].

Another likely impact of climate change is rising food prices [51]. In total, taking into account farming adaptation (varying input use and management practices, and expanding production into new areas) an overall yield reduction of 11% is projected. This is estimated to produce a 20% increase in crop prices but this effect will vary by region and crop type. If food prices rise under climate change then this is a public health concern as rising prices often result in less healthy food choices [52]. Of particular concern is that highly processed foods with high sugar and fat contents (i.e. less healthy foods) are often cheaper than healthier alternatives. More processed food is also less sensitive to food price rises as the cost of the raw ingredients is a smaller component of the total cost. Therefore, increases in food prices may lower the quality of dietary intakes and lower nutritional status. Further impacts of climate change upon the nutritional quality of food are presented elsewhere [1].

### Climate change adaptation

In terms of future risks to food from climate change and how these may be adapted to, it is important to recognise that the chain from farm to fork to possible disease is strictly regulated and monitored to minimise food-borne disease risks in the UK. These provide the UK with resilience against any changes in food-borne disease and highlight where adaptation can occur.

A key example of such regulations is the EU Food Hygiene Regulations (EU, 2004) which set down basic food hygiene rules across the EU which are enforced by member states. In addition to regulations, the monitoring of the levels of disease-causing agents, such as *Salmonella* and *Campylobacter* in food is essential, and across the UK this is the responsibility of a number of different organisations. The monitoring of food quality is important for food produced outside of the EU where the UK has less control on production methods. An example of monitoring leading to improvements in food safety are the voluntary agreements between food producers and the Food Standards Agency against *Salmonella* in eggs [1]. Practical constraints mean that monitoring can only test a tiny fraction of food, highlighting the importance of Hazard Analysis and Critical Control Point type risk assessment along the entire food chain. In the future this could be expanded to identify areas experiencing notable climate change or rapid adaptations by agriculture. In such areas, changes to food-borne disease risks are likely.

The monitoring of human disease associated with food is another important resilience and adaptation mechanism. An example is the report into the deaths from *Salmonella* Typhimurium in 1984 at the Stanley Royd hospital which led to food safety improvements across the UK [53]. Such investigations are increasing in sophistication through, for example, the greater discrimination that whole genome sequencing and other techniques can provide in disease investigation. For example in New Zealand microbial source attribution approaches have been used to target Campyloacter interventions [54]. More problematic are incidences of food borne disease associated with imported food where the UK has less ability to investigate and act. Though the EU wide Rapid Alert System for Food and Feeds, the UK is alerted to food safety issues as they arise within other member states. If changes in food-borne disease are detected then food chain traceability is an essential element to respond to the emerging threat. This is essential because food chains can be complex [55]. Food chain traceability is covered by the EU General Food Law Regulation.

Climate change potentially shifts the weather to new ranges and this could make current regulations and monitoring inadequate. Horizon scanning is one way that such threats could be anticipated. This highlights the importance of groups such as the Human Animal Infections and Risk Surveillance (HAIRS) group which identify and evaluate threats posed by new or re-emerging infectious diseases. This is especially important given the possibility of new stain emergence from animal sources globally. Given the large uncertainties created by climate change, systems such as food early warning systems [56] or food risk detection systems play an important role in responding to climate change induced food threats.

As well as reducing food-borne disease much regulation and monitoring can also benefit the agricultural sector, manufacturers and retailers through reduced costs associated with product recalls and loss of consumer confidence. However, reducing food-borne disease often costs money and it is important to ensure the cost-effectiveness of any interventions.

## Conclusions/evidence gaps

*Campylobacter*, is an important cause of gastrointestinal disease and an increasing public health issue. Although there is reasonable evidence that disease incidence is linked to the environment and weather, uncertainty as to the precise mechanisms makes it difficult to assess the likely impact of climate change. Should climate change increase incidence, and should this follow the current patterns of disease then individuals of higher socioeconomic status and those living in more rural parts of the UK are most likely to be affected. Older and younger individuals are most at risk. Given the uncertainty as to the precise mechanisms through which the environment and weather affect *Campylobacter*, more research is urgently required. Only when there is a much fuller understanding of how climate and weather affect illness with *Campylobacter* will it be possible to make a more complete assessment of the likely impacts of climate change.

*Salmonella* is another important disease examined which exhibits positive associations with ambient temperature. In contrast to *Campylobacter* there is a clear understanding of some of the mechanisms underlying this association. So although climate change may increase disease incidence, because the overall levels of infection with *Salmonella* are declining in the UK these changes are likely to be relatively small. Any changes are likely to affect the young and old disproportionally.

This review has highlighted many pathways through which food may be affected by climate change. However, it has also highlighted that many of these impacts may be indirect and that only a few of these potential impacts have been examined rigorously. Consequently there is a huge degree of uncertainty as to what the overall impact of climate change upon food-borne disease will be.

Given this uncertainty, the resilience of the UK against food-borne disease and the potential to adapt to changes, are of critical importance in a changing world. Agriculture and food processing are highly controlled industries and regular monitoring of food quality and human disease is undertaken. Such information is used to improve public health. Therefore, should climate change alter disease incidence the UK is reasonably resilient and has a capacity to adapt. However, in a new climate regime the ability of current regulations and monitoring to deal with new threats is unknown. This report highlights horizon scanning or real time food early warning systems as potentially useful tools as we move into a more uncertain future, but further research on their efficacy and how they may be enhanced is required.

## Open peer review

Peer review reports for this article are available in Additional file 1.

## Additional file

Additional file 1: Open peer review. (PDF 185 kb)

## Abbreviations

GBS: Guillain-Barre syndrome
HACCP: Hazard Analysis and Critical Control Point
HAIRS: Human Animal Infections and Risk Surveillance
IID: Infectious Intestinal Disease
