# Supplementary material for: Food-borne disease and climate change in the United Kingdom
Source: Environ Health. 2017 Dec 5;16(Suppl 1):117. doi: 10.1186/s12940-017-0327-0 (PMC5773878; doi:10.1186/s12940-017-0327-0)
Supplement: Additional file 1: — Open peer review. (PDF 185 kb) [file 12940_2017_327_MOESM1_ESM.pdf]

Reviewer reports

Title: Food-borne disease and climate change in the United Kingdom

Reviewer 1: James Valcour

The article presented is a review of food-borne disease and climate change. It primarily discusses two pathogens, *Campylobacter jejuni* and *Salmonella* sp. with a geographical focus on impacts for the United Kingdom.

Overall, the review is well presented and covers recent research on the topic. Minor edits and an expanded discussion of some topics would strengthen the overall review of the subject.

The title should be reworded to reflect the focus of the review on the impact of climate change on *Campylobacter* sp. and *Salmonella* sp., either that or the review should be expanded to include other food-borne pathogens, some of which were given a limited examination in the "Other Potential Impacts" section. Likewise, given that the focus is on the United Kingdom, this should also be reflected in the title.

The methodology used to conduct the review should be included. While this article is not a systematic review of the literature, a scoping review of the literature is warranted given the objectives of the review. The inclusion of the search methodology that was used to collect the literature used in the review would provide the reader with an idea as to how comprehensive the literature used in the review is and if a scoping review was indeed conducted.

In the "Other Potential Impacts" section, the focus seems to wander from infectious disease food-borne impacts to more environmental and agricultural impacts. This distracts from the primary message of the review and doesn't add much to the overall thesis of the review.

The inclusion of a section on the potential impacts of global climate change for the United Kingdom would strengthen the review and provide a context for the potential impacts on food-borne disease in the region.

The text needs to distinguish between infections, incidence, and the bacterium. For example, page 3, second paragraph, should read "*Campylobacter* incidence …" as the original wording appears to imply that the bacterium itself "shows strong seasonal variation". This issue regarding wording is prevalent throughout the text, particularly in the section "Climate Change Impacts; *Campylobacter*".

The term "disease burden" requires clarification. The burden of disease is often used to refer to many different measures (e.g. morbidity, mortality, PPYL, DALY). I believe the author is using burden of disease as synonymous with incidence, but it is not clear as the author uses both

terms within the same paragraph. Defining the use of the terminology or using more specific terminology would provide clarity.

The last section that covers evidence gaps should present more specific knowledge gaps that need to be addressed to enhance our understanding of the impact of climate change on food-borne enteric disease incidence.

Declaration of competing interests: I declare that I have no competing interests.

Reviewer 2: Gordon Nichols

Please find enclosed some suggested modifications to the paper by Iain Lake. My overall comments are here:

1. This is a competent and interesting non-systematic review of the impacts that climate change may have on Salmonella and Campylobacter infections, particularly focussing on the UK perspective. It would probably be useful to include this UK focus in the title.
2. The differences between Campylobacter and Salmonella seasonality have been discussed, but further examination of the climate drivers for disease would be useful.
3. In recent years Campylobacter outbreaks have been more common than Salmonella ones. These are mostly associated with chicken liver and weddings. The number of reported Salmonella infections continues to decline and so does the number of outbreaks. However, whole genome sequencing (WHS) is allowing many small clusters of Salmonella to be detected, which can be difficult to follow up. Campylobacter, on the other hand has such large numbers that WGS typing is linked to certain key areas within a research project, because of cost criteria.
4. There is a need to mention intervention through multiple improvements in chicken decontamination. Evidence from action in New Zealand of the effects of cleaning up chicken production and contamination have initiated improvements a public health intervention in the UK. There is monthly monitoring of Campylobacter in the neck skin of chickens from the major supermarkets and publication of results. This has applied pressure to retailers to force chicken producers to take measures to reduce contamination of flocks and FSA / DEFRA have also worked on approaches to reducing contamination of retail chickens. Such interventions appear to be having some impact on case numbers. It is likely that further interventions will have an impact on case numbers, although evidence from New Zealand suggests that the reduction in case numbers will be partial only. This is probably because the routes of transmission are still not fully elucidated.
5. Insect transmission has been suggested as a way that chicken flocks can become contaminated, and there could also be more direct transmission from faeces to dinner plate through this route. It is feasible that cases will decline in the future as a result of such interventions.
6. As a majority of Salmonella cases are travel related in England and Wales, it would be useful to emphasise the likely changes in disease associated with warmer conditions in holiday destinations. This may be particularly relevant to Spain, which is the most visited foreign destination and is likely to be impacted by warmer temperatures.
7. The main Salmonella serotypes/eburst genotypes seen in different countries vary. Based on historical precedent it is likely that at some time in the future there will be emergence of strains from different animal sources across Europe and such emergence is difficult to predict.
8. Future changes resulting from the greater discrimination that Whole Genome Sequencing can provide should be discussed as this will be an additional change with respect to intervention.

I think this paper is suitable for publication in The Journal of Environmental Health with minor changes.

Declaration of competing interest: I declare that I have no competing interests.

Food-borne disease and climate change

Iain R. Lake[+]*

+School of Environmental Sciences, University of East Anglia, Norwich NR4 7TJ, UK

* Author for correspondence (Tel: +44 1603 593744, Fax: +44 1603 591327) email: I.Lake@uea.ac.uk)

Key Words: Climate Change, Food-borne disease, Adaptation, Salmonella, Campylobacter, Gastrointestinal infections, global warming

To be submitted to: Environmental Health

Abstract

This review examined the likely impact of climate change upon food-borne disease in the UK with a specific focus upon *Campylobacter* and *Salmonella*. *Campylobacter* is an important food-borne disease and an increasing public health threat. There is a reasonable evidence base that the environment and weather play a role in its transmission to humans. However, uncertainty as to the precise mechanisms through which weather affects disease, make it difficult to assess the likely impact of climate change. There are strong positive associations between *Salmonella* cases and ambient temperature and a clear understanding of the mechanisms behind this. However, because the incidence of *Salmonella* is declining in the UK any climate change increases are likely to be small. For both *Salmonella* and *Campylobacter* the burden of disease is greatest in older adults and young children. There are many pathways through which climate change may affect food but only a few of these have been rigorously examined. This provides a high degree of uncertainty as to what the impacts of climate change will be. Food is highly controlled at the National and EU level. This provides the UK with resilience to climate change as well as potential to adapt to its consequences.

Introduction and Scope

Climate change may have many impacts upon food [1]. In this review we focus upon two food-borne diseases, *Campylobacter* and *Salmonella*. These are chosen because, in addition to their public heath importance, there is much evidence that they are influenced by existing climate variability especially temperature [2]. Therefore, under a warmer climate, incidence of these infections may change. The purpose of this review is to consider what the likely impacts of climate change will be, as well as to consider the distributional impacts of any changes. In addition the review will also consider in less detail a number of potential impacts which are less well documented in the literature.

Comment [g1]: Single author

Although the geographical focus of this review is the UK, international borders can be crossed by food-borne disease implying that changes in foodborne disease in one country may have consequences in others. For example, of the infectious intestinal disease recorded in the UK (of which food-borne disease is a subset) 8-12% are estimated to have been caught overseas [3]. Furthermore the food supply chain is global and so any impacts of the food supply chain in one country can have impacts elsewhere. Only 53% of the total food consumed in Britain is home grown [4]. Food and drink are also important export markets for the UK and so climate change induced food safety changes in the UK could have global consequences.

The Evidence; *Campylobacter*

In developed countries, including the UK, *Campylobacter* is the most common bacterial cause of diarrhoeal disease. It can cause abdominal pain and severe diarrhoea. Clinical complications include Guillain-Barre syndrome which requires intensive care in some 20% of cases, and can be fatal [5]. Although poultry consumption is widely implicated as a source of *Campylobacter* many other factors are thought to play a role and many features of the disease are difficult to explain (e.g. spring peak). Consequently the epidemiology of *Campylobacter* is complicated [6] and the transmission pathways for a large proportion of cases are unknown [7]. In terms of UK health outcomes following *Campylobacter* infection a recent study [3] estimates that *Campylobacter* is the major bacterial Infectious Intestinal Disease agent in the UK, leading to over 500,000 cases and 80,000 consultations to general practice annually [8]. In 2008 the annual cost of acute *Campylobacter* infection was estimated to be £600 million for England and Wales [9].Reported *Campylobacter* disease also appears to be increasing [10-12]. These increases have been occurring in spite of biosecurity initiatives to exclude *Campylobacter* from poultry flocks [13].

Campylobacter shows a strong seasonal variability leading researchers to believe that it may be affected by climate change. This is coupled with numerous studies indicating that *Campylobacter* infections are associated with climate variability. The most commonly reported factor is a positive association with temperature [2, 14, 15]. However, our understanding of the reasons behind this are limited because unlike other bacteria *Campylobacter* does not multiple outside the gut. For example the response of *Campylobacter* cases to season and weather patterns has been attributed in the literature to several factors such as the cycling of the organisms in natural reservoirs, seasonality of countryside use, and changes in food consumption (e.g. barbecuing associated with warmer weather)[6]. *Campylobacter* transmission to humans is complex ecologically with multiple hosts and transmission pathways [14], and currently is poorly understood.

Comment [g2]: multiply

In terms of where disease burden is highest, elevated incidence in rural areas is a common finding in many [16, 17] but not all studies [18]. In England and Wales the highest incidence is found in rural areas [6]. In Scotland this rural excess was only observed in the under 5s [19]. Strachan et al. [19] were able to attribute *Campylobacter* infections to different sources using Multilocus Sequence Typing. They argue that the major source of infection for young children in urban areas is chicken, whereas for rural children ruminant and other avian sources are of elevated importance.

Studies across the UK indicate that the burden of disease is higher in less deprived areas [6, 20], although because these studies are based upon reported cases of *Campylobacter*, some differences may be due to differential reporting [21]. Gillespie et al [22] found in England and Wales that *Campylobacter* incidence was slightly higher in individual's whose work was often done in an office or other professional environment in comparison to those whose jobs were more manual. However, incidence was highest in people working in semi-routine occupations [23]. This same study found that the burden of *Campylobacter* disease was greatest in the Pakistani population in comparison to the white population. Levels in other ethnic groups such as Indian, Black and Chinese were lower. Turning to gender, this study found that the burden of disease was slightly higher in males than in females, a result confirmed in Scotland [24]. In terms of the age distribution of reported cases the highest burden appears to fall on infants. Incidence then decreases for the ages 2-13 years but rises again until age 22. Incidence then remains relatively constant between ages 22 and 69 before falling from age 70 onwards [22]. Similar distributions are reported in Scotland [24] and Northern Ireland [12]. In terms of trends over time, as the UK population ages the number of reported *Campylobacter* cases has increased in older individuals. However, as well as the absolute number of reports increasing it has also been observed that *Campylobacter* incidence is increasing in older people [6].

The Evidence; *Salmonella*

Infection with *Salmonella* leads to diarrhoea, fever and abdominal cramps, usually 1 – 3 days after the initial infection. Symptoms generally last for 4-6 days but in some individuals the patient may need to be hospitalised. Although there are a number of potential pathways of transmission for *Salmonella*, the consumption of raw or undercooked eggs or poultry are recognised to be of major importance. ~~Several~~ Over two thousand *Salmonella* ~~species (s~~Serotypes~~)~~ have been identified and these have differing routes of transmission. For example *Salmonella* Enteritidis is commonly associated with eggs whereas *Salmonella* Typhimurium is associated with a wider variety of foods [2]. A recent study in England estimates that there are just under 39,000 cases of *Salmonella* a year leading to just over 11,000 GP consultations [8]. This is a large reduction in cases in comparison to the early 1990's. In contrast to *Campylobacter*, *Salmonella* outbreaks are common and so as a disease it is likely to be prominent in public consciousness. Nonetheless *Salmonella* is not a priority pathogen identified by the Food Standards Agency for specific action [25]. Older research focusing upon England and Wales at a time when *Salmonella* incidence was higher, estimated that in 2000 it led to over 8,500 hospital admissions and 119 deaths [NB more estimated deaths than Campylobacter; 26]. It has been estimated that the average

Comment [g3]: In recent years campylobacter outbreaks have been more common than Salmonella ones. These are mostly associated with chicken liver and weddings. The number of reported Salmonella infections continues toi decline and so does the number of outbreaks. However, whole genome sequencing (WHS) is allowing many small clusters of Salmonella to be detected, which can be difficult to follow up. Campylobacter, on the other hand has such large numbers that WGS typing is linked to certain key areas within a research project, because of cost criteria.

cost of a *Salmonella* case is around £1,000 [27]. Multiplying this by the estimated community cases produces a total UK cost of £39million p/a (This assumes that the costs of reported and non-reported cases are similar and so is probably an overestimate).

Salmonella is climate sensitive and infections are more frequent in summer. Stronger evidence emerges from studies indicating that in warm weather Salmonella infections are elevated [15, 28]. Furthermore, there is a clear biological understanding of the mechanisms involved as Salmonella can grow in food kept at ambient temperature [29]. Therefore in a warmer world, Salmonella infections could increase. Across Europe the numbers of cases are currently declining because intervention under the Zoonosis Directive has proved effective through flock testing, the vaccination of animals, increased biosecurity and slaughtering out.

Comment [g4]: Which animals – I think this is mostly chickens and pigs.

In terms of highlighting whether *Salmonella* is higher in rural or urban areas no UK studies have been conducted. No difference has been found in studies in the USA, Germany and France [30-32]. ~~A~~ New Zealand found a higher incidence in rural areas [33]. This lack of association is backed up by recent microbiological work suggesting that local domestic animals (e.g. cows and sheep) are not a major source of *Salmonella* in humans [34]. There are also no UK studies examining the socioeconomic burden of cases. US studies have found lower incidence in areas with poorer educational attainment [35, 36]. However, this contradicts a Canadian study [37]. There are no UK studies examining differentiation between ethnic groups, but in the US minority populations suffer a greater burden of [36, 38]. In terms of the age distribution of cases in England the reported highest incidence was ~~is~~ in the under 4s reducing until age 14. From this point incidence is fairly constant [10]. Similar age distributions are reported in Scotland [39] and Northern Ireland [12]. The increasing use of proton pump inhibitors may increase susceptibility to *Salmonella* [40] and the use of these in older populations is increasing.

Comment [g5]: Salmonella related disease?

Climate Change Impacts; *Campylobacter*

Comment [g6]: There is a need to mention intervention through multiple improvements in chicken decontamination. Evidence from action in New Zealand of the effects of cleaning up chicken production and contamination have initiated improvements a public health intervention in the UK. There is monthly monitoring of Campylobacter in the neck skin of chickens from the major supermarkets and publication of results. This has applied pressure to retailers to force chicken producers to take measures to reduce contamination of flocks and FSA / DEFRA have also worked on approaches to reducing contamination of retail chickens. Such interventions appear to be having some impact on case numbers. It is likely that further interventions will have an impact on case numbers, although evidence from New Zealand suggests that the reduction in case numbers will be partial only. This is probably because the routes of transmission are still not fully elucidated. Insect transmission has been suggested as a way that chicken flocks can become contaminated, and there could also be more direct transmission from faeces to dinner plate through this route. It is feasible that cases will decline in the future as a result of such interventions.

This review has presented evidence that *Campylobacter* is associated with weather; incidence is greater in the summer and during periods of warmer weather incidence is also elevated. Therefore, it would seem logical to assume that climate change would have an impact upon this disease. Although European Infectious Disease experts share a broad agreement that climate change will impact upon *Campylobacter*, this is not the case in the UK [41]. However, this is at odds with other UK sources [e.g. 42] which do suggest a moderate impact. This ambiguity may be due to uncertainty over the exact pathways

through which weather affects incidence. Weather may be associated with *Campylobacter* but we are unsure as to why. Outside the UK there are projections of changes in *Campylobacter* as a result of climate change. Cullen [43] projects increases in *Campylobacter* in Ireland of between 2 and 3%. A study in Montreal forecasts that by 2055, *Campylobacter* could increase 23% [44]. However, given that such studies effectively treat the mechanisms involved as a "black box" it could be argued that these projections are highly uncertain.

Schijven et al., [45] examines the use of a decision support tool for determining the links between *Campylobacter* and climate change. Instead of examining associations between weather and *Campylobacter* they use a Quantitative Microbial Risk Assessment approach and split their analysis into a number of pathogen pathways (drinking water, bathing water, oysters and chicken fillet). Within each pathway a number of models are used to estimate climate change impacts. The results indicate that *Campylobacter* cases associated with poultry consumption are likely to increase under climate change whereas risks associated with the drinking water pathway are likely to decrease due to increased inactivation in higher warmer temperatures.

Climate Change Impacts; *Salmonella*

There are strong links between *Salmonella* and the environment especially ambient temperature. However, in contrast to *Campylobacter* there is a much clearer biological mechanism explaining why higher temperature leads to an elevated incidence of *Salmonella*. At elevated ambient temperatures *Salmonella* reproduction is enhanced. However, in spite of this biological mechanism, UK Infectious Disease experts still do not consider *Salmonella* to be one of the diseases most likely to be affected by climate change [41]. This may be because control measures appear to have substantially reduced the disease burden since the early 1990's to the point where it is not considered a priority pathogen within the UK. There is further evidence that over time the UK is becoming increasingly tolerant to the effects of temperature upon Salmonella infections [21].

Globally there have been some attempts to model future *Salmonella* changes. A recent Australian study estimated by 2050 an extra 4000 – 7000 *Salmonella* cases annually[46]. A second Australian study found that, assuming that all other factors remain constant, salmonellosis might increase 56% by 2050 in South Australia [47]. A recent European study indicated that under the climate change A1B scenario, the number of *Salmonella* cases could increase 9.3 – 16.9% by the 2080's depending upon the level of mitigation.

No specific details are provided for the UK although the study highlights the UK as a country where the largest increase in cases occurs [48].

Climate Change; Other Potential Impacts

Other intestinal infectious diseases vary seasonally or are sensitive to weather. Consequently climate change could affect such diseases. However, currently there is a lack of evidence on which organisms are likely to be affected and what the public health importance of these are. There are also many different mechanisms through which pathogen prevalence changes could occur, such as changing animal husbandry affecting animal to animal transmission, or new weather patterns altering the survival of pathogens in the environment [49]. Therefore, identifying systems and pathogens most likely to be affected is nearly impossible [49]. It is suggested that pathogens with low infective doses are most likely to be affected by climate change (e.g. enteric viruses, *Shigella* spp., enterohemorrhagic *E. coli* strains and parasitic protozoa). Those with significant environmental persistence (e.g. enteric viruses and parasitic protozoa) are also likely to be most affected alongside pathogens with recognised stress tolerance responses to pH and temperature (e.g. enterohemorrhagic *E. coli* and *Salmonella*) [49].

In addition to infectious intestinal disease climate change may have other impacts on food. For example within agriculture one impact may be changes to the seasonal patterns and abundances of pest species and plant diseases both in the UK and globally. Boxall et al., [50] highlight that these changes will lead farmers to alter their use of herbicides, pesticides [51] and fungicides in response. This may alter the levels of these residues in food. In addition to changing farming practices, climate change may also affect the transport of food contaminants. Changing soil properties may affect the bioavailability of heavy metals [50], while more extreme weather could increase the transport of contaminants by flooding [52].

Another likely impact of climate change is rising food prices [53]. In total, taking into account farming adaptation (varying input use and management practices, and expanding production into new areas) an overall yield reduction of 11% is projected. This is estimated to produce a 20% increase in crop prices but this effect will vary by region and crop type. If food prices rise under climate change then this is a public health concern as rising prices often result in less healthy food choices [54]. Of particular concern is that highly processed foods with high sugar and fat contents (i.e. less healthy foods) are often cheaper than healthier alternatives. More processed food is also less sensitive to food price rises as the cost of the raw ingredients is a smaller component of the total cost. Therefore, increases in

food prices may lower the quality of dietary intakes and lower nutritional status. Further impacts of climate change upon the nutritional quality of food are presented elsewhere [1].

Climate Change Adaptation

In terms of future risks to food from climate change and how these may be adapted to, it is important to recognise that the chain from farm to fork to possible disease is strictly regulated and monitored to minimise food-borne disease risks in the UK. These provide the UK with resilience against any changes in food-borne disease and highlight where adaptation can occur.

A key example of such regulations is the EU Food Hygiene Regulations (EU, 2004) which set down basic food hygiene rules across the EU which are enforced by member states. In addition to regulations, the monitoring of the levels of disease-causing agents, such as *Salmonella* and *Campylobacter* in food is essential, and across the UK this is the responsibility of a number of different organisations. The monitoring of food quality is important for food produced outside of the EU where the UK has less control on production methods. An example of monitoring leading to improvements in food safety are the voluntary agreements between food producers and the Food Standards Agency against *Salmonella* in eggs [1]. Practical constraints mean that monitoring can only test a tiny fraction of food, highlighting the importance of Hazard Analysis and Critical Control Point type risk assessment along the entire food chain. In the future this could be expanded to identify areas experiencing notable climate change or rapid adaptations by agriculture. In such areas, changes to food-borne disease risks are likely.

The monitoring of human disease associated with food is another important resilience and adaptation mechanism. An example is the report into the deaths from *Salmonella* Typhimurium in 1984 at the Stanley Royd hospital which led to food safety improvements across the UK [55]. More problematic are incidences of food borne disease associated with imported food where the UK has less ability to investigate and act. Though the EU wide Rapid Alert System for Food and Feeds, the UK is alerted to food safety issues as they arise within other member states. If changes in food-borne disease are detected then food chain traceability is an essential element to respond to the emerging threat. This is essential because food chains can be complex [56]. Food chain traceability is covered by the EU General Food Law Regulation.

Climate change potentially shifts the weather to new ranges and this could make current regulations and monitoring inadequate. Horizon scanning is one way that such threats could be anticipated. This highlights the importance of groups such as the Human Animal Infections and Risk Surveillance (HAIRS) group which identify and evaluate threats posed by new or re-emerging infectious diseases. Given the large uncertainties created by climate change systems such as food early warning systems [57] or food risk detection systems [58] play an important role in responding to climate change induced food threats.

As well as reducing food-borne disease much regulation and monitoring can also benefit the agricultural sector, manufacturers and retailers through reduced costs associated with product recalls and loss of consumer confidence. However, reducing food-borne disease often costs money and it is important to ensure the cost-effectiveness of any interventions.

Conclusions / Evidence Gaps

Campylobacter, is an important cause of gastrointestinal disease and an increasing public health issue. Although there is reasonable evidence that incidence is linked to the environment and weather, uncertainty as to the precise mechanisms makes it difficult to assess the likely impact of climate. Should climate change increase incidence, and should this follow the current patterns of disease then individuals of higher socioeconomic status and those living in more rural parts of the UK are most likely to be affected. Older and younger individuals are most at risk. Given the uncertainty as to the precise mechanisms through which the environment and weather affect *Campylobacter*, more research is urgently required.

Salmonella is another important disease examined which exhibits positive associations with ambient temperature. In contrast to *Campylobacter* there is a clear understanding of some of the mechanisms underlying this association. So although climate change may increase incidence, because the incidence of *Salmonella* is declining in the UK these changes are likely to be relatively small. Any changes are likely to affect the young and old disproportionally.

This review has highlighted many pathways through which food may be affected by climate change. However, it has also highlighted that many of these impacts may be indirect and that only a few of these potential impacts have been examined rigorously. Consequently there is a huge degree of uncertainty as to what the overall impact of climate change upon food-borne disease will be.

The UK has a built in resilience against food-borne disease and a reasonable capacity to adapt to any changes in food safety associated with climate change. Agriculture and food processing are highly controlled industries and regular monitoring of food quality and human disease is undertaken. Such information is used to improve public health. Therefore, should climate change alter disease incidence the UK is reasonably resilient and has a capacity to adapt. However, in a new climate regime the ability of current regulations and monitoring to deal with new threats is unknown. This report highlights horizon scanning or real time food early warning systems as useful tools as we move into a more uncertain future.

List of Abbreviations used

GBS - Guillain-Barre syndrome

IID - Infectious Intestinal Disease

HACCP - Hazard Analysis and Critical Control Point

HAIRS - Human Animal Infections and Risk Surveillance

Competing Interests

None

Acknowledgements

I gratefully acknowledge Living with Environmental Change for funding this review. Dr Lake is part funded by The National Institute for Health Research, Health Protection Research Unit in Emergency Preparedness and Response at King's College London and also the National Institute for Health Research, Health Protection Research Unit in Gastroenterology at Liverpool University.

References

1. Lake IR, Hooper L, Abdelhamid A, Bentham G, Boxall ABA, Draper A, Fairweather-Tait S, Hulme M, Hunter PR, Nichols G *et al*: **Climate change and food security: Health impacts in developed countries**. *Environmental Health Perspectives* 2012, **120**(11):1520-1526.
2. Lake IR, Gillespie IA, Bentham G, Nichols GL, Lane C, Adak GK, Threlfall EJ: **A re-evaluation of the impact of temperature and climate change on foodborne illness**. *Epidemiology and Infection* 2009:1-10.
3. Food Standards Agency: **The Second Study of Infectious Intestinal Disease in the Community (IID2 Study)**. In. London: Food Standards Agency; 2011.
4. DEFRA: **Food Statistics Pocketbook 2013 - in year update**. In. London: Department for Environment Food and Rural Affairs; 2013.
5. World Health Organisation: **The Global View of Campylobacteriosis; Report of an Expert Consultation**. In. Utrecht, Netherlands, 9-11 July 2012; 2012.

6. Nichols GL, Richardson JF, Sheppard SK, Lane C, Sarran C: **Campylobacter epidemiology: A descriptive study reviewing 1 million cases in England and Wales between 1989 and 2011**. *BMJ Open* 2012, **2**(4).
7. Tam CC, Rodriguez LC, O'Brien SJ, Hajat S: **Temperature dependence of reported Campylobacter infection in England, 1989-1999**. *Epidemiology and Infection* 2006, **134**(1):119-125.
8. Tam CC, Rodrigues LC, Viviani L, Dodds JP, Evans MR, Hunter PR, Gray JJ, Letley LH, Rait G, Tompkins DS *et al*: **Longitudinal study of infectious intestinal disease in the UK (IID2 study): Incidence in the community and presenting to general practice**. *Gut* 2012, **61**(1):69-77.
9. BBSRC, FSA, DEFRA, Scottish Government: **UK Research and Innovation Strategy for Campylobacter in the food chain 2010-2015**. In. London: HMSO; 2010.
10. Health Protection Agency: **Gastrointestinal infections annual report, 2010**. In. London: Health Protection Agency; 2011.
11. Health Protection Scotland: **Campylobacter, Scotland, Annual Totals** In.; 2013.
12. Public Health Agency: **Epidemiology of Gastrointestinal Infections in Northern Ireland; Annual Surveillance Report 2012**. In.; 2013.
13. Allen VM, Newell DG: **Food standards agency report commissioned project ms0004; Evidence for the effectiveness of biosecurity to exclude Campylobacter from poultry flocks**. In. London: Food Standards Agency; 2005.
14. Kovats RS, Edwards SJ, Charron D, Cowden J, D'Souza RM, Ebi KL, Gauci C, Gerner-Smidt P, Hajat S, Hales S *et al*: **Climate variability and campylobacter infection: An international study**. *International Journal of Biometeorology* 2005, **49**(4):207-214.
15. Naumova EN, Jagai JS, Matyas B, DeMaria A, MacNeill IB, Griffiths JK: **Seasonality in six enterically transmitted diseases and ambient temperature**. *Epidemiology and Infection* 2007, **135**(2):281-292.
16. Levesque S, Fournier E, Carrier N, Frost E, D. Arbeit R, Michaud S: **Campylobacteriosis in urban versus rural areas: A case-case study integrated with molecular typing to validate risk factors and to attribute sources of infection**. *PLoS ONE* 2013, **8**(12).
17. Pasturel BZ, Cruz-Cano R, Goldstein RER, Palmer A, Blythe D, Ryan P, Hogan B, Jung C, Joseph SW, Wang MQ *et al*: **Impact of rurality, broiler operations, and community socioeconomic factors on the risk of campylobacteriosis in Maryland**. *American Journal of Public Health* 2013, **103**(12):2267-2275.
18. Spencer SEF, Marshall J, Pirie R, Campbell D, Baker MG, French NP: **The spatial and temporal determinants of campylobacteriosis notifications in New Zealand, 2001-2007**. *Epidemiology and Infection* 2012, **140**(9):1663-1677.
19. Strachan NJC, Gormley FJ, Rotariu O, Ogden ID, Miller G, Dunn GM, Sheppard SK, Dallas JF, Reid TMS, Howie H *et al*: **Attribution of campylobacter infections in northeast scotland to specific sources by use of multilocus sequence typing**. *Journal of Infectious Diseases* 2009, **199**(8):1205-1208.
20. MacRitchie LA, Hunter CJ, Strachan NJC: **A population-based exposure assessment of risk factors associated with gastrointestinal pathogens: A Campylobacter study**. *Epidemiology and Infection* 2013, **141**(5):976-986.
21. Lake IR, Nichols G, Harrison FCD, Bentham G, Sari Kovats R, Grundy C, Hunter PR: **Using infectious intestinal disease surveillance data to explore illness aetiology; a cryptosporidiosis case study**. *Health and Place* 2009, **15**(1):333-339.
22. Gillespie IA, O'Brien SJ, Penman C, Tompkins D, Cowden J, Humphrey TJ: **Demographic determinants for Campylobacter infection in England and Wales: Implications for future epidemiological studies**. *Epidemiology and Infection* 2008, **136**(12):1717-1725.

23. Chandola T, Bartley M, Wiggins R, Schofield P: **Social inequalities in health by individual and household measures of social position in a cohort of healthy people**. *Journal of Epidemiology and Community Health* 2003, **57**(1):56-62.
24. Smith-Palmer A, Cowden J: **The epidemiology of Campylobacter infection in Scotland**. In.: Health Protection Scotland; 2008.
25. Food Standards Agency: **Foodborne Disease Strategy 2010-2015; An FSA Programme for the reduction of foodborne disease in the UK**. In. Lonon: Food Standards Agency; 2011.
26. Adak GK, Meakins SM, Yip H, Lopman BA, O'Brien SJ: **Disease Risks from Foods, England and Wales, 1996–2000**. *Emerging Infectious Diseases* 2005, **11**(3).
27. Santos AC, Roberts JA, Cook AJC, Simons R, Sheehan R, Lane C, Adak GK, Clifton-Hadley FA, Rodrigues LC: **Salmonella Typhimurium and Salmonella Enteritidis in England: Costs to patients, their families, and primary and community health services of the NHS**. *Epidemiology and Infection* 2011, **139**(5):742-753.
28. Kovats RS, Edwards SJ, Hajat S, Armstrong BG, Ebi KL, Menne B, Cowden J, Gerner-Smidt P, Herna?ndez Pezzi G, Kristufkova Z *et al*: **The effect of temperature on food poisoning: A time-series analysis of salmonellosis in ten European countries**. *Epidemiology and Infection* 2004, **132**(3):443-453.
29. D'Souza RM, Becker NG, Hall G, Moodie KBA: **Does ambient temperature affect foodborne disease?** *Epidemiology* 2004, **15**(1):86-92.
30. Arshad MM, Wilkins MJ, Downes FP, Rahbar MH, Erskine RJ, Boulton ML, Younus M, Saeed AM: **Epidemiologic attributes of invasive non-typhoidal Salmonella infections in Michigan, 1995-2001**. *International Journal of Infectious Diseases* 2008, **12**(2):176-182.
31. Delarocque-Astagneau E, Bouillant C, Vaillant V, Bouvet P, Grimont PAD, Desenclos JC: **Risk factors for the occurrence of sporadic Salmonella enterica serotype typhimurium infections in children in France: A national case-control study**. *Clinical Infectious Diseases* 2000, **31**(2):488-492.
32. Karsten C, Baumgarte S, Friedrich AW, Von Eiff C, Becker K, Wosniok W, Ammon A, Bockemühl J, Karch H, Huppertz HI: **Incidence and risk factors for community-acquired acute gastroenteritis in north-west Germany in 2004**. *European Journal of Clinical Microbiology and Infectious Diseases* 2009, **28**(8):935-943.
33. Lal A, Baker MG, French NP, Dufour M, Hales S: **The epidemiology of human salmonellosis in New Zealand, 1997-2008**. *Epidemiology and Infection* 2012, **140**(9):1685-1694.
34. Mather AE, Reid SWJ, Maskell DJ, Parkhill J, Fookes MC, Harris SR, Brown DJ, Coia JE, Mulvey MR, Gilmour MW *et al*: **Distinguishable epidemics of multidrug-resistant Salmonella typhimurium DT104 in different hosts**. *Science* 2013, **341**(6153):1514-1517.
35. Younus M, Hartwick E, Siddiqi AA, Wilkins M, Davies HD, Rahbar M, Funk J, Saeed M: **The role of neighborhood level socioeconomic characteristics in Salmonella infections in Michigan (1997-2007): Assessment using geographic information system**. *International Journal of Health Geographics* 2007, **6**.
36. Chang M, Groseclose SL, Zaidi AA, Braden CR: **An ecological analysis of sociodemographic factors associated with the incidence of salmonellosis, shigellosis, and E. coli O157:H7 infections in US counties**. *Epidemiology and Infection* 2009, **137**(6):810-820.
37. Varga C, Pearl DL, McEwen SA, Sargeant JM, Pollari F, Guerin MT: **Evaluating area-level spatial clustering of Salmonella Enteritidis infections and their socioeconomic determinants in the greater Toronto area, Ontario, Canada**

(2007 - 2009): A retrospective population-based ecological study. *BMC Public Health* 2013, **13**(1).
38. Quinlan JJ: **Foodborne illness incidence rates and food safety risks for populations of low socioeconomic status and minority race/ethnicity: a review of the literature**. *International journal of environmental research and public health* 2013, **10**(8):3634-3652.
39. Health Protection Scotland and Food Standards Agency (Scotland): **Joint Annual Report on Infectious Intestinal Disease Associated With Foodborne Illness in Scotland**. In.: Health Protection Scotland; 2013.
40. Leonard J, Marshall JK, Moayyedi P: **Systematic review of the risk of enteric infection in patients taking acid suppression**. *American Journal of Gastroenterology* 2007, **102**(9):2047-2056.
41. Semenza JC, Suk JE, Estevez V, Ebi KL, Lindgren E: **Mapping climate change vulnerabilities to infectious diseases in Europe**. *Environmental Health Perspectives* 2012, **120**(3):385-392.
42. North West Public Health Observatory: **The impact of Climate Change upon Health and Health Inequalities in the North West of England**. In.; 2012.
43. Cullen E: **The Impact of Climate Change on the Future Incidence of Specified Foodborne Diseases in Ireland**. *Epidemiology* 2009, **60**(6):S227-S228.
44. Allard R, Plante C, Garnier C, Kosatsky T: **The reported incidence of campylobacteriosis modelled as a function of earlier temperatures and numbers of cases, Montreal, Canada, 1990-2006**. *International Journal of Biometeorology* 2011, **55**(3):353-360.
45. Schijven J, Bouwknegt M, de Roda Husman AM, Rutjes S, Sudre B, Suk JE, Semenza JC: **A Decision Support Tool to Compare Waterborne and Foodborne Infection and/or Illness Risks Associated with Climate Change**. *Risk Analysis* 2013, **33**(12):2154-2167.
46. Bambrick H, Dear K, Woodruff R, Hanigan I, McMichael A: **Garnaut Climate Change Review; The impacts of climate change on three health outcomes: temperature-related mortality and hospitalisations, salmonellosis and other bacterial gastroenteritis, and population at risk from dengue**. In.; 2008.
47. Zhang Y, Bi P, Hiller JE: **Projected burden of disease for Salmonella infection due to increased temperature in Australian temperate and subtropical regions**. *Environment International* 2012, **44**(1):26-30.
48. Kovats S, Lloyd S, Hunt A, Watkiss P (eds.): **Technical Policy Briefing Note 5: The Impacts and Economic Costs on Health in Europe and the Costs and Benefits of Adaptation, Results of the EC RTD ClimateCost Project.** . Sweden: Stockholm Environment Institute; 2011.
49. FAO: **Climate Change: Implications for Food Safety**. In. Rome: Food and Agriculture Organisation; 2008.
50. Boxall ABA, Hardy A, Beulke S, Boucard T, Burgin L, Falloon PD, Haygarth PM, Hutchinson T, Kovats RS, Leonardi G *et al*: **Impacts of Climate Change on Indirect Human Exposure to Pathogens and Chemicals from Agriculture**. *Environmental Health Perspectives* 2009, **17**(4):508-514.
51. Hirschi M, Stoeckli S, Dubrovsky M, Spirig C, Calanca P, Rotach MW, Fischer AM, Duffy B, Samietz J: **Downscaling climate change scenarios for apple pest and disease modeling in Switzerland**. *Earth System Dynamics* 2012, **3**(1):33-47.
52. Lake IR, Foxall CD, Fernandes A, Lewis M, Rose M, White O, Lovett AA, White S, Dowding A, Mortimer D: **The effects of flooding on dioxin and PCB levels in food produced on industrial river catchments**. *Environment International* 2015, **77**:106-115.
53. Nelson GC, Valin H, Sands RD, Havlík P, Ahammad H, Deryng D, Joshua Elliottf, Fujimori S, Hasegawa T, Heyhoe E *et al*: **Climate change effects on agriculture:**

Economic responses to biophysical shocks. *Proceedings of the National Academy of Sciences of the United States of America* 2014, **111**(9):3274–3279.

54. Cummins S, Macintyre S: **Food environments and obesity-neighbourhood or nation?** *International Journal of Epidemiology* 2006, **35**:100-104.
55. Hugill J: **The Report of the Committee of Inquiry into an Outbreak of Food Poisoning at Stanley Royd Hospital, Cmnd 9716.** In. London: HMSO; 1986.
56. Food Standards Agency: **Update on investigation into horse and pig DNA in beef products; 16th January 2013**. In.; 2013.
57. Marvin HJP, Kleter GA, Prandini A, Dekkers S, Bolton DJ: **Early identification systems for emerging foodborne hazards**. *Food and Chemical Toxicology* 2009, **47**(5):915-926.
58. Groeneveld R, Willems D, Broekstra J, van den Broek W, Top J: **ERDSS: Emerging Risk Detection Support System.** In. Wageningen: Agrotechnology and Food Sciences Group; 2008.

Author response

Dear Professor Ebi,

Thank you for providing us with the opportunity to perform minor corrections on the paper that I have written entitled:

Food-borne disease and climate change

I attach a revised version of the paper incorporating all the comments of the reviewers and editors. I also enclose with this letter a note detailing our responses to the reviewers' comments (reviews comments are underlined).

If you require any further information please do not hesitate to contact me.

Yours sincerely,

Dr. Iain Lake

Reviewer 1:

The article presented is a review of food-borne disease and climate change. It primarily discusses two pathogens, *Campylobacter jejuni* and *Salmonella* sp. with a geographical focus on impacts for the United Kingdom. Overall, the review is well presented and covers recent research on the topic. Minor edits and an expanded discussion of some topics would strengthen the overall review of the subject.

I thank the reviewer for these positive comments on the paper.

The title should be reworded to reflect the focus of the review on the impact of climate change on *Campylobacter* sp. and *Salmonella* sp., either that or the review should be expanded to include other food-borne pathogens, some of which were given a limited examination in the "Other Potential Impacts" section.

Much of the review focussed upon Salmonella and Campylobacter. These were chosen because there has been much research on them but also because they are exemplar organisms that provide insight into how climate change may affect a rage of food-borne diseases. I have strengthened the section in the paper describing why they were chosen but also kept the broad title of the paper to reflect this.

Likewise, given that the focus is on the United Kingdom, this should also be reflected in the title.

I agree and have changed the title to include "United Kingdom"

The methodology used to conduct the review should be included. While this article is not a systematic review of the literature, a scoping review of the literature is warranted given the objectives of the review. The inclusion of the search methodology that was used to collect the literature used in the review would provide the reader with an idea as to how comprehensive the literature used in the review is and if a scoping review was indeed conducted.

I have reworded the introduction to make it clear that this was not a systematic review. I have additionally given further information on the chronology of the document to enable a fuller understanding of the methodology.

In the "Other Potential Impacts" section, the focus seems to wander from infectious disease food-borne impacts to more environmental and agricultural impacts. This distracts from the primary message of the review and doesn't add much to the overall thesis of the review.

The purpose of the review was to be all encompassing. Hence I feel that although this section is relatively small, it covers a range of other, often unresearched or considered issues relating to climate change. Hence, I feel it important that this section remains. It contains references for readers wanting to explore these in further detail.

The inclusion of a section on the potential impacts of global climate change for the United Kingdom would strengthen the review and provide a context for the potential impacts on food-borne disease in the region.

As this is a special issue focussing upon climate change in the UK this will be a common issue for all the papers. Hence I have simply included a reference to the "Health Impacts of Climate Change in the UK report" where the reader may obtain more specific information about the likely impact of climate change upon the UK.

The text needs to distinguish between infections, incidence, and the bacterium. For example, page 3, second paragraph, should read "*Campylobacter* incidence …" as the original wording

appears to imply that the bacterium itself "shows strong seasonal variation". This issue regarding wording is prevalent throughout the text, particularly in the section "Climate Change Impacts; *Campylobacter*".

Again I thank the referee for highlight this inconsistency in my terminology. I have been through the paper and ensured consistency throughout.

The term "disease burden" requires clarification. The burden of disease is often used to refer to many different measures (e.g. morbidity, mortality, PPYL, DALY). I believe the author is using burden of disease as synonymous with incidence, but it is not clear as the author uses both terms within the same paragraph. Defining the use of the terminology or using more specific terminology would provide clarity.

Again I thank the referee for highlight this inconsistency in my terminology. I have been through the paper and ensured consistency throughout.

The last section that covers evidence gaps should present more specific knowledge gaps that need to be addressed to enhance our understanding of the impact of climate change on food-borne enteric disease incidence.

I thank the reviewer for highlighting these gaps in my conclusions. A fuller set of evidence gaps has now been provided.

Reviewer 2:

This is a competent and interesting non-systematic review of the impacts that climate change may have on Salmonella and Campylobacter infections, particularly focussing on the UK perspective. It would probably be useful to include this UK focus in the title.

This is similar to the comment for reviewer 1 and the title has been changed accordingly.

The differences between Campylobacter and Salmonella seasonality have been discussed, but further examination of the climate drivers for disease would be useful.

Reviewing this section we have added a few sentences to the climate drivers for Campylobacter but in my opinion this was done sufficiently for Salmonella.

In recent years Campylobacter outbreaks have been more common than Salmonella ones. These are mostly associated with chicken liver and weddings. The number of reported Salmonella infections continues to decline and so does the number of outbreaks. However, whole genome sequencing (WHS) is allowing many small clusters of Salmonella to be detected, which can be difficult to follow up. Campylobacter, on the other hand has such large numbers that WGS typing is linked to certain key areas within a research project, because of cost criteria.

There does not seem to be an obvious comment to respond to here.

There is a need to mention intervention through multiple improvements in chicken decontamination. Evidence from action in New Zealand of the effects of cleaning up chicken production and contamination have initiated improvements a public health intervention in the UK. There is monthly monitoring of Campylobacter in the neck skin of chickens from the major supermarkets and publication of results. This has applied pressure to retailers to force chicken producers to take measures to reduce contamination of flocks and FSA / DEFRA have also worked on approaches to reducing contamination of retail chickens. Such interventions appear to be having some impact on case numbers. It is likely that further interventions will have an impact on case numbers, although evidence from New Zealand suggests that the reduction in

case numbers will be partial only. This is probably because the routes of transmission are still not fully elucidated.

Insect transmission has been suggested as a way that chicken flocks can become contaminated, and there could also be more direct transmission from faeces to dinner plate through this route. It is feasible that cases will decline in the future as a result of such interventions.

As suggested we have strengthened the section on interventions with new material

As a majority of Salmonella cases are travel related in England and Wales, it would be useful to emphasise the likely changes in disease associated with warmer conditions in holiday destinations. This may be particularly relevant to Spain, which is the most visited foreign destination and is likely to be impacted by warmer temperatures.

I am unclear where this figure has come from because the official data suggests that this proportion is lower at around a quarter. Nevertheless I have added a sentence acknowledging this.

The main Salmonella serotypes/eburst genotypes seen in different countries vary. Based on historical precedent it is likely that at some time in the future there will be emergence of strains from different animal sources across Europe and such emergence is difficult to predict.

This has been added into the paper

Future changes resulting from the greater discrimination that Whole Genome Sequencing can provide should be discussed as this will be an additional change with respect to intervention.

This has been added into the paper
